# Editorial: Mechanisms of microenvironment governed plasticity and progression in solid tumors

**DOI:** 10.3389/fcell.2024.1373496

**Published:** 2024-03-25

**Authors:** Jonathan A. Kelber, Marcin Iwanicki, Marianna Kruithof-de Julio, Benjamin T. Spike, Michelle M. Martínez-Montemayor

**Affiliations:** ^1^ California State University, Northridge, Los Angeles, United States; ^2^ Department of Biology, Baylor University, Waco, TX, United States; ^3^ Stevens Institute of Technology, Hoboken, NJ, United States; ^4^ Department of Urology, Inselspital, Bern University Hospital, University of Bern, Bern, Switzerland; ^5^ Urology Research Laboratory, Department for BioMedical Research, University of Bern, Bern, Switzerland; ^6^ Department of Oncological Sciences, The University of Utah, Salt Lake City, UT, United States; ^7^ School of Medicine, Huntsman Cancer Institute, The University of Utah, Salt Lake City, UT, United States; ^8^ Central University of the Caribbean, Bayamón, Puerto Rico

**Keywords:** cancer plasticity, tumor heterogeneity, microenvironment, multiomics, single-cell analyses, mechanobiology

## Introduction

The growth, spread and persistence of transformed cancer cells involves their aberrant interaction with various microenvironments that result in tumor cell plasticity and intratumoral heterogeneity. A comprehensive understanding of tumor progression therefore requires not only an in-depth examination of cancer cells but also an exploration of tumor microenvironments (TMEs) and the reciprocity between them. In this Research Topic collection, original studies describe TME components, including emphases on the identification of new markers for cancer-associated fibroblasts (CAFs) and mechanical cues that support signal transduction driving cancer stem cells. Additionally, two review articles present contemporary insights into the role of extracellular vesicles in cancer progression and the mechanisms governing breast cancer metastasis.

Kazakova et al. utilize publicly available single-cell RNA-sequencing data from multiple solid tumor types and corresponding normal tissues, the researchers identified novel CAF markers. They uncovered CAF-specific gene expression signatures and identified 10 protein markers showing strong positive staining in tumor stroma based on IHC images in the Human Protein Atlas database. This study demonstrates innovative computational approaches to characterize TME components and identifies crucial markers of activated CAFs across different solid tumor types. Rosado-Galindo et al. employ substrate micropatterning on polystyrene films, the researchers investigated the effect of topographical cues on the proteome and stemness of TNBC cell lines. Substrate surface roughness enriched cancer stem cells (CSCs) and modulated epidermal growth factor receptor (EGFR) signaling activity. The study reported phenotypic changes associated with topographically rough stimuli, indicating a potential modulation of the response to the EGFR inhibitor gefitinib. CD44+/CD24−/ALDH + cells and YAP/TAZ signaling were enhanced on rough surface substrates, suggesting an important role for substrate topography in modulating responses to EGFR inhibition, and CSC enrichment in TNBC models.

Lopez et al. provide a comprehensive review that explores extracellular vesicles (EVs) in the context of solid tumor progression. The authors summarize various EV isolation and characterization approaches needed for an accurate understanding of their association with diseases. They delve into different EV subclasses, methods for isolation and characterization, and highlight current clinical trials studying EVs. The review also covers key studies exploring the role and impact of EVs in the TME, detailing how EVs mediate intercellular communication, drive cancer progression, and remodel the TME. Si et al. This perspective review argues that the prolonged latency period between initial treatment and eventual recurrence in breast cancer patients indicates the ability of tumors to adapt to and interact with the systemic host environment, facilitating and sustaining disease progression. The authors stress the need for a comprehensive framework surrounding the mechanisms driving the growth, survival, and spread of tumor cells, encompassing tumor cell interaction with supporting cells in the microenvironment. Recent discoveries concerning critical aspects of breast cancer metastasis, including the intricate network of cells, molecules, and physical factors contributing to metastasis are considered, along with an exploration of the molecular mechanisms governing cancer dormancy.

## Future perspectives

Deconstructing, understanding and intervening in tumor cell-TME crosstalk is a key goal for the advancement of cancer treatment. Emerging technologies promise to enhance and facilitate our understanding of cancer cell interactions within various TMEs. Integrating spatial transcriptomics with proteome analysis and image/AI-based topographical resolution of malignant and normal tissues at cellular resolution will provide a more comprehensive view of cancer and its TMEs. However, a major challenge remains in faithfully reconstructing these data and systems in next-generation cancer models to facilitate studying the molecular mechanisms of disease progression and personalizing intervention strategies.

