# Correction: Editorial: Mechanisms of microenvironment governed plasticity and progression in solid tumors

**DOI:** 10.3389/fcell.2025.1702303

**Published:** 2025-09-23

**Authors:** Jonathan A. Kelber, Marcin Iwanicki, Marianna Kruithof-de Julio, Benjamin T. Spike, Michelle M. Martínez-Montemayor

**Affiliations:** ^1^ California State University, Northridge, Los Angeles, CA, United States; ^2^ Department of Biology, Baylor University, Waco, TX, United States; ^3^ Stevens Institute of Technology, Hoboken, NJ, United States; ^4^ Department of Urology, Inselspital, Bern University Hospital, University of Bern, Bern, Switzerland; ^5^ Urology Research Laboratory, Department for BioMedical Research, University of Bern, Bern, Switzerland; ^6^ Department of Oncological Sciences, The University of Utah, Salt Lake City, UT, United States; ^7^ School of Medicine, Huntsman Cancer Institute, The University of Utah, Salt Lake City, UT, United States; ^8^ Central University of the Caribbean, Bayamón, Puerto Rico

**Keywords:** cancer plasticity, tumor heterogeneity, microenvironment, multiomics, single-cell analyses, mechanobiology

The funder National Institutes of Health-National Institute of General Medical Sciences (NIGMS) and Department of Defense were erroneously omitted. The correct funding statement reads:

“The author(s) declare that financial support was received for the research and/or publication of this article. This work was supported by National Institutes of Health-National Institute of General Medical Sciences (NIGMS) grant number R16GM145488 to M-M and National Institutes of Health-National Institute of General Medical Sciences (NIGMS) grant number SC1GM121182 to JAK and by the Department of Defense Breast Cancer Research Program (DoD-BCRP) grant number W81XWH-21-1-0801 to BTS.”

The original article has been updated.

